# One-Year Results of Simultaneous Topography-Guided Photorefractive Keratectomy and Corneal Collagen Cross-Linking in Keratoconus Utilizing a Modern Ablation Software

**DOI:** 10.1155/2015/321953

**Published:** 2015-08-23

**Authors:** A. M. Sherif, M. A. Ammar, Y. S. Mostafa, S. A. Gamal Eldin, A. A. Osman

**Affiliations:** Department of Ophthalmology, Faculty of Medicine, Cairo University, Cairo 12411, Egypt

## Abstract

*Purpose.* To evaluate effectiveness of simultaneous topography-guided photorefractive keratectomy and corneal collagen cross-linking in mild and moderate keratoconus.* Methods*. Prospective nonrandomized interventional study including 20 eyes of 14 patients with grade 1-2 keratoconus that underwent topography-guided PRK using a Custom Ablation Transition Zone (CATz) profile with 0.02% MMC application immediately followed by standard 3 mw/cm^2^ UVA collagen cross-linking. Maximum ablation depth did not exceed 58 *μ*m. Follow-up period: 12 months.* Results.* Progressive statistically significant improvement of UCVA from 0.83 ± 0.37 logMAR preoperative, reaching 0.25 ± 0.26 logMAR at 12 months (*P* < 0.001). Preoperative BCVA (0.27 ± 0.31 logMAR) showed a progressive improvement reaching 0.08 ± 0.12 logMAR at 12 months (*P* = 0.02). Mean *K*max reduced from 48.9 ± 2.8 to 45.4 ± 3.1 D at 12 months (*P* < 0.001), mean *K*min reduced from 45.9 ± 2.8 D to 44.1 ± 3.2 D at 12 months (*P* < 0.003), mean keratometric asymmetry reduced from 3.01 ± 2.03 D to 1.25 ± 1.2 D at 12 months (*P* < 0.001). The safety index was 1.39 at 12 months and efficacy index 0.97 at 12 months.* Conclusion*. Combined topography-guided PRK and corneal collagen cross-linking are a safe and effective option in the management of mild and moderate keratoconus.* Precis*. To our knowledge, this is the first published study on the use of the CATz ablation system on the Nidek Quest excimer laser platform combined with conventional cross-linking in the management of mild keratoconus.

## 1. Introduction

Keratoconus is a chronic bilateral noninflammatory corneal degeneration, characterized by localized corneal thinning and corneal steepening, leading to irregular astigmatism and impaired visual acuity [1].

Corneal collagen cross-linking has found a broad international application for keratoconus in recent years [2, 3]. The combination of ultraviolet A (UVA) light with riboflavin as a photosensitizing agent produces interfibrillar cross-linking between corneal stromal collagen fibers, thus increasing corneal rigidity and halting the ectatic process. The spherocylindrical change that it causes is limited [4].

Several adjuvant therapies in combination with CXL have been proposed in an effort to develop a technique that can offer patients keratectasia corneal stability together with improved functional vision, including intracorneal rings [5] and phakic intraocular lenses [6].

Topography-guided PRK to correct ametropia and irregular astigmatism in form fruste and frank keratoconus was introduced over a decade ago [7–9] with good results. However, concerns were raised about the long-term complications, being a tissue subtraction technique.

The mechanism of topography-guided ablation is the fitting of an ideal corneal shape (usually a sphere) under the present topography map with the ablation of tissue in between.

Topography-guided PRK flattens not only some of the cone peaks but also an arcuate broader area of the cornea away from the cone, usually in the superior nasal periphery; this ablation pattern will resemble part of a hyperopic treatment and thus will cause some amount of steepening, or elevation adjacent to the cone, effectively normalizing the cornea [10].

Kanellopoulos and Binder were the first to combine CXL with topography-guided PRK in the management of keratoconus. They introduced a two-step procedure with CXL performed first and topography-guided PRK after a 1-year interval [2].

However, the fact that cross-linked corneas may have a different ablation rate from normal corneas—which could lead to unpredictable PRK results—and the hypothesis that the removal of the cross-linked corneal tissue by the PRK procedure could decrease the stiffening effects of the CXL treatment in addition to the increased possibility of haze formation after PRK were serious limitations [3].

Since then, several studies have evaluated the simultaneous use of topography-guided PRK followed immediately by CXL in progressive keratoconus [9, 11–13].

The aim of this study is to evaluate the effectiveness of combined corneal collagen cross-linking and topography-guided PRK in cases of mild and moderate keratoconus using an advanced ablation software with the Quest laser platform (Nidek, Gamagori, Japan).

## 2. Materials and Methods

This nonrandomized prospective clinical study was conducted on 20 eyes of 14 Caucasian patients in between February 2012 and December 2013. The study was conducted within the tenets of the Declaration of Helsinki after the approval of the institutional review board. A written informed consent was obtained from all patients.

All cases were performed at Eye Care Center for refractive surgery, Cairo, Egypt.

Inclusion criteria included grade 1 or 2 stable keratoconus (Amsler-Krumeich classification) [10] documented by topography with a corneal thickness not less than 450 *μ*m and above 18 years of age. Exclusion criteria included patients below 18 years of age, advanced (stage 3 or 4 Amsler-Krumeich classification), BSCVA worse than 1 logMAR, central corneal scars, cases that underwent previous refractive surgery, and patients with history of herpetic eye disease or who were pregnant or lactating.

Preoperative examination included complete ocular examination included the following: assessment of uncorrected visual acuity (UCVA) and best spectacle corrected visual acuity (BSCVA), slit-lamp examination, intraocular pressure (IOP) measurement using Goldman's applanation tonometry, assessment of manifest and cycloplegic refraction, and fundus examination using indirect ophthalmoscopy.

Corneal topography was performed using Optical Path Difference (OPD scan II) scanning System (Nidek, Gamagori, Japan). The device collects corneal topography, ocular wavefront, autorefraction, and pupillometric data. The topography is Placido-based, collecting data from more than 6800 points. Axial map data were collected. Measurements were repeated at least 3 times, and the best image was chosen for the final analysis (the best one was being defined as the image with the best quality peaks for individual points). Soft contact lenses were stopped one week before topography while rigid gas permeable and hard contact lenses were stopped 2 weeks before topography.

Corneal pachymetry was done using PacScan 300P ultrasound pachymeter (Sonomed Escalon, NY, USA) with map mode to measure 5 points at the cornea (centre, superior, inferior, temporal, and nasal area). Measurement accuracy and repeatability are assured by each scan actually consisting of 256 individual measurements and an automatic measurement algorithm to ensure that only scans with proper probe alignment are accepted.

Surgeries were performed by 2 surgeons (Y. S. Mostafa and A. M. Sherif).

### 2.1. Operative Steps

**Step 1 (topography-guided PRK).**

After topical anaesthesia, the epithelium was mechanically removed within an 8.5 mm diameter using hockey knife. Topography-guided PRK was performed with the aim to normalize the cornea, by reducing irregular astigmatism and also treating part of the refractive error. Custom Ablation Transition Zone (CATz) ablation profile (Quest, Nidek, Japan) was used. The CATz algorithm delivers aspheric treatment zones combined with the treatment of corneal elevation irregularities or corneal wavefront at the surgeon's choice. The treatment was planned using the Final Fit software version 1.11T3,4 (Nidek, Co., Ltd.) based on the curvature and elevation maps from the Placido disc system of the linked topography device OPD scan II.

To ensure minimal tissue removal, the effective optical zone diameter was decreased to 5.5 mm. The transition zone was 1.5 mm. Correction of up to 70% of cylinder in addition to up to 40% of the spherical component—without exceeding a maximal ablation depth limit of 50 *μ*m—was attempted. Corneal target asphericity was set to −0.5 and no tilt correction was attempted. An example of the ablation pattern is shown in Figure 6.

After completion of ablation, 0.02% mitomycin C was applied for 30 seconds, followed by copious irrigation with balanced salt solution.

**Step 2 (corneal collagen cross-linking).**

For the next 20 minutes, 0.1% riboflavin 5-phosphate plus 20% Dextran T 500 ophthalmic solution (Ribolink Isotonic, Optos, Australia) was applied topically every 2 minutes. The solution appeared to “soak” into the corneal stroma rapidly, as it was centrally devoid of Bowman's layer. Following the initial riboflavin administration, collagen cross-linking was performed by projecting Ultraviolet (UV) light at 370 nm wavelength (365 to 375 nm) and 3 mW/cm^2^ radiance at 4.5 cm onto the surface of the cornea for 30-minute X link cross-linking system (Opto Global, Australia). The device has an accurate internal power meter and feedback loop control deliver consistent UV irradiation during the entire procedure and eliminates the need for periodic calibration.

A bandage contact lens was placed on the cornea at the completion of the procedure and was removed at 3–5 days following complete reepithelialization.

Postoperative treatment included the topical antibiotic moxifloxacin (Vigamox, Alcon) four times a day for the first week and topical nonsteroidal anti-inflammatory drops Nepafenac 0.1% (Nevanac, Alcon Research Ltd., Fort Worth, TX) for 3 to 5 days until complete epithelial healing. This was followed by an antibiotic/steroid combination (Tobradex, Alcon) to be tapered over 60 days. 1000 mg Vitamin C was given orally for 30 days.

Postoperative examinations were performed by 2 independent observes (M. A. Ammar and A. A. Osman).

Patients were assessed 1 month, 3 months, 6 months, and 12 months after surgery. On each visit, the patients were examined for uncorrected visual acuity (UCVA), best corrected visual acuity (BCVA), epithelial healing, and haze formation according to Fantes haze grading system using slit lamp [14] and corneal topography was performed using OPD scan II. Total eye aberrometry was not recorded in all cases and thus was not included in the results. Examples of pre- and postoperative topography maps are shown in Figure 5.

### 2.2. Statistical Methods

Data management and analysis were performed using Statistical Package for Social Sciences (SPSS) version 17.

Data were summarized using means and standard deviations. To examine the changes between the different time periods for normally distributed variables, a one-way repeated measures analysis of variance was conducted. For nonnormally distributed variables, the analyses were performed by the Friedman test, a nonparametric repeated measures one-way analysis of variance. Pairwise comparisons were done using the Wilcoxon Signed test after adjusting the *P* values using the Bonferroni corrections. *P* values ≤0.05 were considered significant.

The main outcome measures of the study were UCVA and BCVA.

Secondary outcome measures were *K*max, *K*min, and keratometric asymmetry. The data were obtained from the axial maps of the OPD scan.

In addition, the safety and efficacy indices were calculated [15].

The predictability index was not included because we were not sure about the fine accuracy of the subjective spherical equivalent and because the aim was more of surface regularization rather than spherocylindrical correction.

## 3. Results

The study included 20 eyes of 14 patients: 11 eyes belonged to males (55%) and 9 eyes belonged to females (45%). The age of patients ranged from 18 to 29 years with a mean of 23.6 ± 3.2.

The results are summarized in Table 1.

The mean ablation depth was 48.09 ± 9.6 *μ*m (range 23.3–57.3 *μ*m).

The mean target cylindrical correction was 1.25 ± 0.85 D (range 0–3.25 D).

The preoperative and 1-, 3-, 6-, and 12-month postoperative sphere, cylindrical, and spherical equivalent data are shown in Table 2.

**Figure 1:**
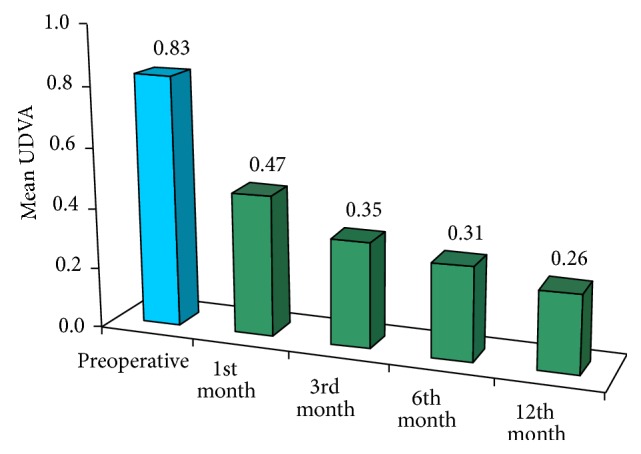
Preoperative and postoperative uncorrected distance visual acuity.

There was a statistically significant difference between the mean preoperative logMAR UCVA (0.83 ± 0.37) (0.21 ± 0.19 decimals) and 1-month postoperative logMAR UCVA (0.47 ± 0.23) (0.41 ± 0.27 decimals) (*P* < 0.001). UCVA continued to improve progressively until the end of the 12-month follow-up period (0.25 ± 0.26 logMAR) (0.63 ± 0.25 decimals) (*P* < 0.001) shown in Figure 1.

**Figure 2:**
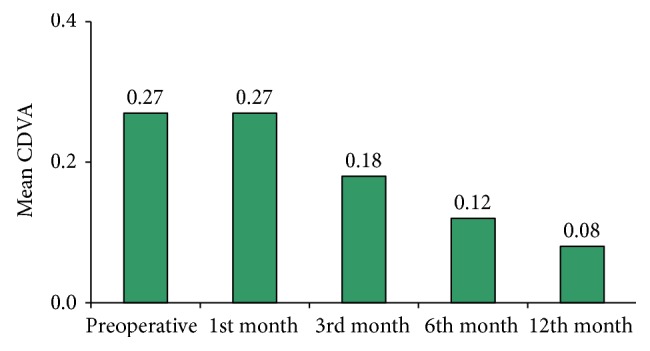
Preoperative and postoperative best corrected distance visual acuity.

The preoperative mean logMAR BCVA (0.27 ± 0.31) (0.62 ± 0.29 decimals) showed an insignificant change (*P* = 0.99) one month postoperatively (0.27 ± 0.29 logMAR) (0.61 ± 0.28 decimal) and continued to improve over the follow-up period, reaching 0.18 ± 0.21 logMAR at 3 months (*P* = 0.108), 0.12 ± 0.19 logMAR (0.82 ± 0.29 decimal) at 6 months (*P* = 0.04), and 0.08 ± 18 logMAR (0.89 ± 0.27 decimal) at 12 months (*P* = 0.02) (as shown in Figure 2).

**Figure 3:**
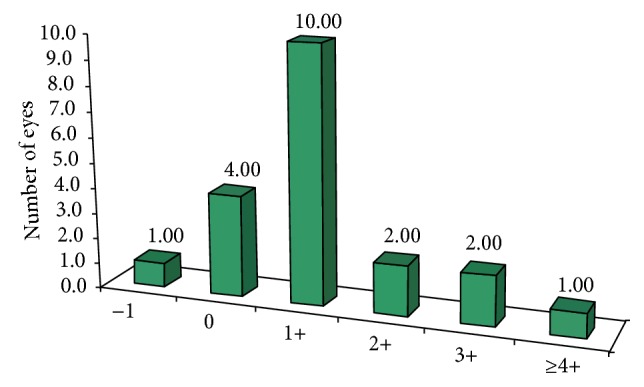
Gain/loss of lines of CDVA.

Two eyes (20%) showed no improvement in BCVA in lines, 10 eyes (50%) gained one line, 10% gained two lines, 10% gained 3 lines, and one eye (5%) gained 4 lines in BCVA. One eye (5%) lost one line in BCVA at the 12th month follow-up in comparison to preoperative BCVA due to grade 3 haze (as shown in Figure 3).

The efficacy index (postoperative mean decimal UDVA divided by preoperative mean decimal BCDVA) was 0.66 one month after surgery, improved to 0.88 at 6 months, and continued to improve at 12 months, reaching 0.97.

The safety index (postoperative mean decimal BCDVA divided by preoperative mean decimal BCDVA) showed a progressive improvement from 0.96 at one month, reaching 1.31 at 6 months and settling at 1.39 at 12 months.

**Figure 4:**
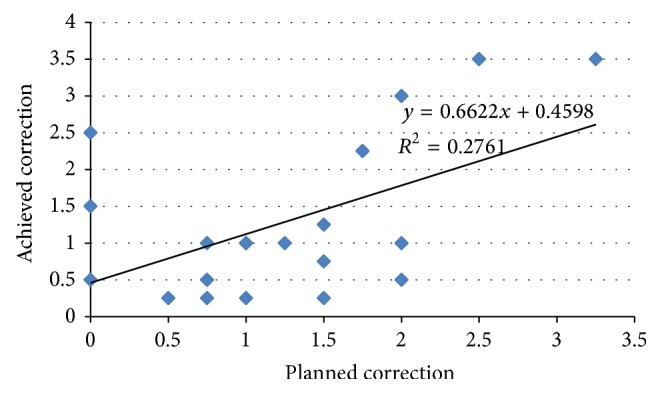
Linear regression analysis graph of planned and achieved cylindrical correction.

**Figure 5:**
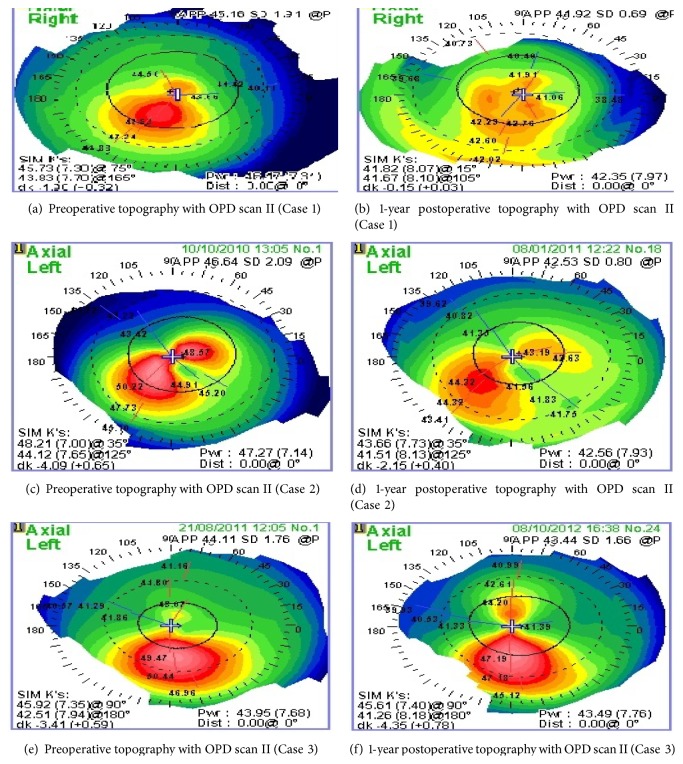
Comparative topography maps before and after surgery.

**Figure 6:**
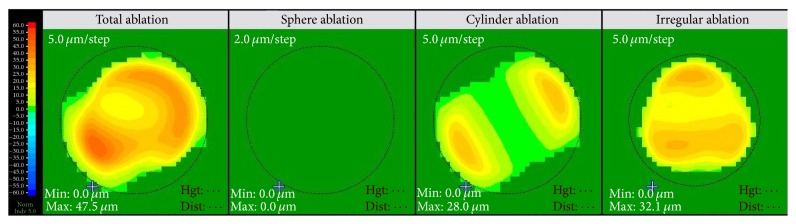
The ablation profile in CATz (Quest, Nidek excimer laser machine).

The mean of planned cylindrical correction was 1.25 ± 0.85 D and the mean achieved cylindrical correction was 1.29 ± 1.08 D at 12 months (as shown in Figure 4).

The median of the preoperative cylinder was reduced from 2.13 D to 1.25 D at one month (*P* < 0.001). It remained stable over the follow-up period (1 D at 3, 6 months (*P* < 0.001) and 1.13 D at 12 months (*P* < 0.001)).

The mean keratometric asymmetry was reduced from 3.01 ± 2.03 D preoperatively to 1.25 ± 1.2 D at 12 months (*P* < 0.001).

The improvement in mean *K*max and mean *K*min was stable over the 12-month follow-up period as shown in Table 1.

No serious complications were recorded during the follow-up period. 30% of cases had mild haze 1 month after surgery that was gradually reduced with topical steroids, reaching 20% at 3 months, 10% at 6 months, and 5% at 12 months.

## 4. Discussion

This study evaluated simultaneous topography-guided PRK and corneal CXL for treatment of early cases of keratoconus.

It has been possible to use topography-guided excimer laser treatments in highly irregular corneas that are beyond the limits of wavefront measuring devices, making this approach more efficient in treating highly irregular astigmatism, such as in keratoconus, as its measurements are based solely on the cornea surface reflection [10].

Sequential CXL-Topo-guided PRK one year apart was first introduced to address the refractive element of keratoconus using PRK and the biomechanical aspect using CXL [2]. However several later studies reported better results with simultaneous Topo PRK and CXL regarding UCVA, BCVA, keratometry reduction, and haze [3, 16].

In our study, the preoperative mean UCVA was 0.83 ± 0.37 which improved at the 1st month postoperatively to 0.47 ± 0.32 with a *P* value of <0.001 which was statistically significant. It continued to improve over the following months, reaching 0.26 ± 0.25 at the last follow-up (12th month).

These results are better than the results of Lin et al. [10] in 2012 and comparable to the results reported by Alessio et al. in 2013 [11], Mukherjee et al. in 2013 [12], and Kanellopoulos in 2009 [3] and slightly worse than the results of Kymionis et al. in 2009 [17].

The preoperative mean BCVA was 0.27 ± 0.31 which showed a gradual improvement over the follow-up period reaching 0.12 ± 0.19 (*P* = 0.04) at 6 months. At the last follow-up (12th month) the mean BCVA was 0.08 ± 0.18 (*P* = 0.02).

20% of eyes in our study showed no improvement in BCVA in Snellen chart lines, 50% of eyes gained one line, 10% gained two lines, 10% gained 3 lines, and one eye (5%) gained 4 lines in BCVA.

These results are comparable to the results reported by Kymionis et al. [17], Kanellopoulos [3], and Stojanovic et al. in 2010 [13].

While one eye (5%) lost one line in BCVA at the 12th month postoperative follow-up in comparison to preoperative BCVA, Lin et al. reported that 12.5% of eyes lost 1 line and 4% lost >2 lines of BCVA [10]. However, Alessio et al. [11] and Mukherjee et al. [12] reported no loss of lines of BCVA.

The reduction in mean* K*max in our study was around 3.3 D, similar to the results of Kymionis et al. [17], Chan et al. [5], Alessio et al. [11], and Mukherjee et al. [12].

Regarding corneal haze, only one eye (5%) had visually significant haze (grade 3). Eight eyes (40%) had trace haze (grade 1) and 55% of eyes had no haze at all at the end of the 12-month follow-up. This concurs with other studies on simultaneous collagen cross-linking and topography-guided PRK [16, 18].

Previous studies [3, 11–13, 16–18] evaluated the technique of simultaneous topography-guided PRK with CXL in keratoconus with encouraging results and few postoperative complications.

To our knowledge, there are no previously published studies describing the use of the CATz software of the Quest Nidek excimer laser system for topography-guided PRK in combination with collagen cross-linking in keratoconus. Our results showed significant progressive improvement of UCVA and BCVA and low risk of haze throughout the 12-month follow-up period.

One of the limitations of the study was that the maximum ablation depth of 50 *μ*m was exceeded in two cases (53.5 *μ*m and 57.3 *μ*m). In addition, rigid gas permeable and hard contact lenses were stopped 2 weeks only before surgery which may be too short for some corneas. In addition, the follow-up period in our study was 12 months and due to the reports of progressive excessive corneal flattening [19, 20] which may lead to hyperopic shift up to several years after CXL [21], further studies with longer follow-up periods are needed to further evaluate the long-term outcomes of this technique.

## Figures and Tables

**Table 1:** Summary of results.

Parameter	Preoperatively	1 month postoperatively	3 months postoperatively	6 months postoperatively	12 months postoperatively
LogMAR UDVA Mean ± SD	0.830 ±.37	0.47 ±0.32	0.35 ±0.25	0.31 ±0.26	0.26 ±0.25
*P *value		**0.003**	**<0.001**	**<0.001**	**<0.001**

LogMAR CDVA Mean ± SD	.27 ±0.31	0.27 ±0.29	0.18 ±0.21	0.12 ±0.19	0.08 ±0.18
*P *value		0.99	0.108	**0.04**	0.02

*K*max in D Mean ± SD	48.9 ±2.8	45.6 ±3.2	45.6 ±3.2	45.5 ±3.1	45.4 ±3.1
*P *value		**<0.001**	**<0.001**	**<0.001**	**<0.001**

*K*min in D Mean ± SD	45.9 ±2.8	44.4 ±2.9	44.3 ±3.0	44.3 ±3.1	44.1 ±3.2
*P *value		0.004	**0.003**	0.003	0.003

Median cylindrical error in D	2.13	1.25	**1.0**	1.00	**1.13**
*P *value		**<0.001**	**<0.001**	**<0.001**	**<0.001**

LogMAR = logarithm of the minimal angle of resolution, UDVA = uncorrected distance visual acuity, CDVA = corrected distance visual acuity, *K*max = maximum keratometry, *K*min = minimum keratometry, and D = diopter.

**Table 2:** Pre- and postoperative sphere, cylindrical and spherical equivalent values.

	Sph. preoperative	Sph. 1 m	Sph. 3 m	Sph. 6 m	Sph. 12 m	Cyl. preoperative	Cyl. 1 m	Cyl. 3 m	Cyl. 6 m	Cyl. 12 m	SE preoperative	SE 1 m	SE 3 m	SE 6 m	SE 12 m
Case 1	1.5	0.75	0.5	0.5	0.5	1.5	0.75	0.75	0.5	0.5	2.25	1.13	0.88	0.75	0.75
Case 2	2.5	1	1	0.75	0.75	1.5	1	0.5	0.5	0.5	3.25	1.5	1.25	1	1
Case 3	3	1.5	1.5	1.5	1.5	2.25	1.5	1.25	1	1.25	4.13	2.25	2.13	2.13	2.13
Case 4	1	0.25	0	0	0	1.5	0.75	0.75	1	0.5	1.75	0.63	0.37	0.5	0.25
Case 5	2.5	1.75	1.5	1.25	1	8.5	6.75	6	5.5	5.5	6.75	5.13	4.5	4	3.75
Case 6	1.5	0.25	0.5	0.25	0.25	2.75	1.5	2	2.25	2	2.88	1	1.5	1.38	1.25
Case 7	1.5	0.75	0.75	0.5	0.5	1.75	0.75	1	0.75	0.5	2.38	1.13	1.25	0.88	0.75
Case 8	1.75	0.75	0.5	0.5	0.5	0.75	1.25	0.5	0.5	0.5	2.13	1.38	0.75	0.75	0.75
Case 9	2	0.75	0.75	1	1	1.5	1	0.75	1.25	1.25	2.75	1.25	1.13	1.63	1.63
Case 10	0.75	0.75	0.75	0.5	0.75	6.5	3.25	5	4.5	4	4	2.38	3.25	3	2.75
Case 11	1	0.75	0.75	0.75	0.75	4	4	4	3.75	3.5	3	2.75	2.75	2.63	2.25
Case 12	0.5	0.5	0.5	0.5	0.5	2	1	0.75	0.5	0.5	1.5	1	0.87	0.75	0.75
Case 13	1.75	0.75	0.75	0.5	0.5	0.75	1	0.5	0.5	0.25	2.13	1.25	1	0.75	0.63
Case 14	1.5	1	1	1	1	2.75	1.5	1	1	0.5	2.88	1.75	1.5	1.5	1.25
Case 15	1.25	0.75	1	0.75	0.75	1.5	1	1.25	1	0.75	2	1.25	1.63	1.25	1.12
Case 16	1	0.75	0.5	0.5	0.5	2.5	2	2.25	1.75	2	2.25	1.75	1.63	1.5	1.68
Case 17	2	1.5	1.25	1.25	1	3	2.5	2.5	2.25	2	3.5	2.75	2.5	2.38	2
Case 18	0.5	0.25	0	0	0.25	4.75	1.25	1.25	1	1.25	2.63	0.88	0.68	0.5	0.68
Case 19	0.75	0.25	0.25	0.5	0.25	0.75	0.75	0.5	0.5	0.5	1.13	0.63	0.5	0.75	0.5
Case 20	1.25	1	1	0.75	0.75	4.5	1.25	1	1.25	1	3.5	1.63	1.5	1.38	1.25

Sph.: sphere, cyl.: cylinder, and SE: spherical equivalent; all are in minus dioptric form.
